# Impact of E-health Literacy on Diabetes Self-Care Activities Among People With Type 2 Diabetes Attending Primary Healthcare Centers in Makkah City, Saudi Arabia: A Cross-Sectional Study, 2025

**DOI:** 10.7759/cureus.87968

**Published:** 2025-07-15

**Authors:** Waleed S AlShehri, Yasser Y Khojah

**Affiliations:** 1 Public Health, Makkah Health Cluster, Makkah, SAU; 2 Infection Prevention and Control, King Fahad General Hospital, Jeddah, SAU

**Keywords:** diabetes self-care, digital health, e-health literacy, glycemic control, saudi arabia, type 2 diabetes mellitus

## Abstract

Background: The rising prevalence of type 2 diabetes mellitus (T2DM) in Saudi Arabia necessitates effective self-management strategies. E-health literacy, the ability to access and utilize digital health information, may play a critical role in improving diabetes self-care. This study examined the relationship between e-health literacy and self-care activities among patients with T2DM in Makkah City.

Methodology: A cross-sectional study was conducted with 360 patients with T2DM attending primary healthcare centers in Makkah. Participants completed a structured questionnaire assessing sociodemographic characteristics, e-health literacy, and diabetes self-care activities. Hierarchical regression and logistic regression analyses were performed to evaluate associations while controlling for confounders.

Results: The mean e-health literacy score was moderate (3.13/5), with significant gaps in evaluating online health resources. Higher e-health literacy was strongly associated with improved self-care practices (β = 0.497, *P* < 0.001) and better glycemic control (odds ratio (OR) = 2.985, *P* < 0.001). Key barriers included difficulty understanding medical terms (205, 56.9%), distrust in online sources (196, 54.4%), and lack of time (220, 61.1%). Significant disparities were observed, with males, younger individuals, and those with higher education and income reporting greater e-health literacy.

Conclusions: E-health literacy significantly influences diabetes self-care and glycemic outcomes among patients with T2DM in Saudi Arabia. However, barriers such as limited digital skills and distrust in online health information hinder optimal utilization. Targeted interventions to enhance e-health literacy, particularly for older, less educated, and female patients, are essential for improving diabetes management. Future research should explore culturally tailored digital health education programs to bridge these gaps.

## Introduction

The increasing prevalence of type 2 diabetes mellitus (T2DM) globally [1], particularly in regions such as the Gulf Cooperation Council (GCC) countries [2], necessitates a closer examination of self-care practices among affected individuals. In Saudi Arabia, where T2DM is a significant public health concern [3], the role of e-health literacy in enhancing diabetes self-care activities is gaining attention. E-health literacy refers to the ability to effectively use information technology (IT) to enhance health outcomes [4]. E-health literacy encompasses the skills required to effectively navigate and utilize electronic health resources, which can significantly influence patients' ability to manage their condition.

Research indicates that e-health literacy has a direct impact on self-care behaviors among diabetic patients. It was established that individuals with higher mobile e-health literacy exhibited improved self-care practices, including adherence to medication regimens and lifestyle modifications necessary for effective diabetes management [4,5]. This relationship underscores the importance of equipping patients with the necessary digital skills to access health information and support services online.

Saudi Arabia, with 68.5% of its population having internet access, ranks among the highest in the Middle East in terms of connectivity. Younger Saudis, in particular, increasingly rely on information technology to access health information [6]. Healthcare practitioners and institutions also leverage the internet as a vital resource. This high access and usability of the internet led to the increasing adoption of e-health interventions in the management and prevention of T2DM in Saudi Arabia [7]. Numerous studies highlight the use of e-health and health IT for prevention, treatment, health maintenance, and wellness [7,8]. Mobile applications, for example, guide managing chronic conditions such as cardiovascular diseases and diabetes. Established platforms like WebMD and the US Centers for Disease Control and Prevention (CDC) offer comprehensive health education [9]. Additionally, health-specific portals, such as the Diabetes Center, demonstrate the transformative impact of IT on diabetes management.

Studies have shown that e-health literacy can bolster patients' confidence in managing their health, leading to improved self-care behaviors [10]. Diabetes self-care activities are multifaceted and include monitoring blood glucose levels, dietary management, physical activity, and medication adherence [11,12]. These activities are crucial for preventing complications associated with diabetes. However, many patients struggle with these self-management tasks due to various barriers, including limited health literacy and lack of access to reliable health information [5]. Limited health literacy adversely affects health knowledge, preventive behaviors, and adherence to care plans [13,14]. The integration of e-health resources can bridge this gap by providing patients with tailored information and tools that enhance their understanding of diabetes management.

Understanding the impact of e-health literacy on diabetes self-care activities is vital for developing effective educational programs and interventions. The healthcare system in Saudi Arabia is evolving toward incorporating digital health solutions; however, there remains a need for comprehensive training programs that enhance both health literacy and digital skills among patients [15]. Despite advancements in digital health technologies, many diabetic patients also remain unaware of available resources or lack the skills necessary to use them effectively [16,17]. This gap highlights a critical area for intervention. By assessing the current levels of e-health literacy among diabetic patients and their correlation with self-care practices, healthcare providers can identify specific educational needs and develop targeted interventions. Such initiatives could include training programs that enhance both digital skills and health literacy, thereby empowering patients to take control of their diabetes management. By fostering an environment where patients are empowered to utilize e-health resources effectively, healthcare providers can improve overall health outcomes for individuals living with T2DM.

This study aimed to investigate the relationship between e-health literacy and diabetes self-care activities among individuals with T2DM attending primary healthcare centers in Makkah City, Saudi Arabia. The objectives included assessing levels of e-health literacy, identifying barriers to accessing and using digital health information, and determining how e-health literacy impacts adherence to self-care behaviors such as diet, physical activity, blood glucose monitoring, and foot care. Additionally, the study sought to explore how sociodemographic and clinical factors influence e-health literacy and how these, in turn, affect glycemic control and patients’ confidence in managing their diabetes.

## Materials and methods

Study setting

The study was conducted in primary healthcare centers (PHCs) located in Makkah City, Saudi Arabia. As a major metropolitan area, Makkah offers diverse healthcare services, including comprehensive diabetes management programs at PHCs. These centers are critical points of care for diabetes patients, providing preventive, curative, and educational services.

Study design

This study used an analytical cross-sectional study design. This approach enables the simultaneous collection of data on e-health literacy levels, diabetes self-care activities, and related health outcomes, providing a snapshot of the relationship between these variables among the target population.

Target population

The study targeted patients with T2DM who regularly attended selected PHCs in Makkah, Saudi Arabia. Participants met the following inclusion criteria: Saudi nationals aged 18 years or older who had attended at least two follow-up visits at the selected PHCs and had used online health information resources. Non-Saudi individuals and those under 18 years old were excluded to ensure cultural and contextual relevance in assessing e-health literacy.

Sampling strategy

The minimum required sample size was calculated to be 350 participants using G*Power version 3.1.7 software (Universität Kiel, Kiel, Germany). The calculation was based on an effect size of 0.60, a 5% margin of error, 80% statistical power, and a 95% confidence interval. A stratified sampling technique was employed to ensure representativeness. Makkah was divided into seven administrative regions, and one PHC from each region was randomly selected. Within each PHC, participants were recruited using systematic random sampling. Specifically, every second eligible patient visiting the PHC during the study period was approached for inclusion until the required sample of 50 participants per center was achieved.

Data collection methods and tools

Data were collected using a structured questionnaire developed by the researcher after a thorough review of relevant literature. The questionnaire was also administered during face-to-face interviews, supplemented by a review of participants’ medical records.

The questionnaire comprises the following sections.

Sociodemographic Information

This section gathered details on participants’ age, gender, marital status, educational level, employment status, and monthly income.

Medical History and Data

Information collected included the duration of diabetes, presence of comorbidities, diabetic complications, type of treatment (oral medications or insulin), smoking status, body mass index (BMI), physical activity levels, depressive symptoms, self-reported health status, and recent laboratory results (fasting blood sugar (FBS) and lipid profiles). HbA1c values were primarily obtained from participants’ medical records. Missing data were handled using listwise deletion for regression analyses, excluding cases with incomplete key variables to maintain the validity of statistical models.

E-health Literacy Assessment

E-health literacy was assessed using the Saudi E-health Literacy Scale (eHEALS), a validated tool consisting of eight items. Participants rated their responses on a 5-point Likert scale, ranging from *Strongly Disagree* (1) to *Strongly Agree* (5). The total score was calculated to derive a mean e-health literacy score. The scores were also categorized as follows: 1-2 = low, 3 = moderate, 4-5 = high.

Diabetes Self-Care Activities Assessment

Diabetes self-care activities were measured using the Arabic version of the Summary Diabetes Self-Care Activities (SDSCA) scale [18]. This validated tool includes eight items divided into four domains: diet, physical activity, self-monitoring of blood glucose, and foot care. Participants will report the frequency of specific self-care activities performed over the past week.

Data collection tool validity

The questionnaire was reviewed by a panel of experts in diabetes care, e-health, and public health. The expert panel comprised clinicians, public health specialists, and digital health researchers, who provided feedback on the cultural appropriateness, clarity, and relevance of items. 

To ensure that the questionnaire is culturally appropriate and accurately translated into Arabic, the questionnaire was translated from English to Arabic by a bilingual expert. A second bilingual expert then translated the Arabic version back into English. The translated questionnaire was then reviewed by a panel of experts to ensure that the questions were culturally appropriate for the Saudi population. Any culturally sensitive or inappropriate questions were modified. The translated questionnaire will be pilot-tested with a small sample of Arabic-speaking participants to ensure that the questions are clear and culturally relevant, and the results will be used to refine the questions. 

Statistical analysis

Statistical analyses were performed using SPSS version 25.0 (IBM Corp., Armonk, NY). Descriptive statistics, including frequencies, percentages, means, and standard deviations, summarized the socio-demographic and clinical characteristics of participants, as well as their e-health literacy and self-care activity scores. For inferential analyses, univariate methods such as t-tests, chi-square tests, and correlation analyses were used to assess the relationships between individual variables (e.g., sociodemographic factors, e-health literacy) and diabetes self-care activities. To account for potential confounders such as age, gender, education, and disease duration, multivariate linear regression models were applied to evaluate the independent association between e-health literacy and self-care behaviors. Additionally, logistic regression was used to analyze the impact of e-health literacy on health outcomes, such as achieving target HbA1c levels. A *P*-value of <0.05 was considered statistically significant throughout the analysis.

Ethical considerations

The Ethics Committee approved this study before data collection began. Participants were informed about the study’s purpose, procedures, potential risks, and benefits, and provided written informed consent. Participant anonymity and data confidentiality were strictly maintained. Personal identifiers were not recorded, and all data were securely stored and accessible only to the research team.

## Results

Sociodemographic and clinical characteristics of study participants

Table 1 presents the sociodemographic and clinical characteristics of the 360 participants. The age distribution of participants indicated a mature population; the largest proportion fall within the 50-59 age group (*n* = 95, 26.4%), closely followed by the 40-49 age group (*n* = 87, 24.2%) and those aged 60 and above (*n* = 86, 23.9%). Individuals younger than 30 years constituted the smallest group (*n* = 32, 8.9%). The study sample was predominantly male (*n* = 282, 78.3%), with females representing a smaller proportion (*n* = 78, 21.7%). Most participants were married (*n* = 258, 71.7%), while single, divorced, and widowed individuals accounted for 56 (15.6%), 21 (5.8%), and 25 (6.9%), respectively.

**Table 1:** Sociodemographic and clinical characteristics of study participants (N = 360).

Characteristic	Category	n	%
Age (years)	<30	32	8.9
	30-39	60	16.7
	40-49	87	24.2
	50-59	95	26.4
	60 and above	86	23.9
Gender	Male	282	78.3
	Female	78	21.7
Marital status	Single	56	15.6
	Married	258	71.7
	Divorced	21	5.8
	Widowed	25	6.9
Educational level	No formal education	20	5.6
	Primary education	28	7.8
	Secondary education	68	18.9
	College/University degree	186	51.7
	Postgraduate education	58	16.1
Employment status	Employed (full-time)	197	54.7
	Employed (part-time)	29	8.1
	Unemployed	53	14.7
	Retired	81	22.5
Monthly household income (SAR)	<3,000	42	11.7
	3,000-5,000	52	14.4
	5,001-10.000	84	23.3
	>10.000	182	50.6
Region of residence	City Center	297	82.5
	Suburban area	43	11.9
	Rural area	20	5.6
Duration of diabetes	<1 year	55	15.3
	1-5 years	95	26.4
	6-10 years	72	20
	>10 years	138	38.3
Type of diabetes treatment	Oral medications only	221	61.4
	Insulin only	91	25.3
	Combination of oral medications and insulin	48	13.3
Presence of comorbidities	Hypertension	223	61.9
	Hyperlipidemia	130	36.1
	Cardiovascular disease	65	18.1
	Kidney disease	20	5.6
	Others	1	0.3
	None	117	32.5
Diabetic complications experienced	Neuropathy	117	32.5
	Retinopathy	83	23.1
	Foot ulcers	41	11.4
	Others (Khawmol)	1	0.3
	None	213	59.2
Smoking status	Never smoked	214	59.4
	Former smoker	53	14.7
	Current smoker	93	25.8
Physical activity level	Sedentary	114	31.7
	Light activity	134	37.2
	Moderate activity	105	29.2
	Vigorous activity	7	1.9

Regarding educational attainment, more than half of the participants held a college or university degree (*n* = 186, 51.7%), and a significant portion also had postgraduate education (*n* = 58, 16.1%). Conversely, 20 (5.6%) reported no formal education. In terms of employment, the majority were employed full-time (*n* = 197, 54.7%), followed by retired individuals (*n* = 81, 22.5%). A notable proportion of participants (*n* = 182, 50.6%) reported a monthly household income of above 10,000 SAR. The vast majority of participants resided in the City Center (*n* = 297, 82.5%), reinforcing the urban focus of the study.

The duration of diabetes varied among participants; the largest group had lived with diabetes for more than 10 years (*n* = 138, 38.3%). A substantial portion (*n* = 95, 26.4%) had diabetes for one to five years. The most common type of diabetes treatment reported was oral medications only (*n* = 221, 61.4%), while a quarter of the participants used insulin only (*n* = 91, 25.3%).

Comorbidities were prevalent within the study population; hypertension was the most frequently reported co-existing condition (*n* = 223, 61.9%), followed by hyperlipidemia (*n* = 130, 36.1%) and cardiovascular disease (*n* = 65, 18.1%). Approximately one-third of the participants (*n* = 117, 32.5%) reported no comorbidities. Regarding diabetic complications, neuropathy was the most common (*n* = 117, 32.5%), followed by retinopathy (*n* = 83, 23.1%) and foot ulcers (*n* = 41, 11.4%). Despite these reported complications, a significant majority (*n* = 213, 59.2%) reported no complications.

In terms of lifestyle factors, most participants reported never having smoked (*n* = 214, 59.4%), although a quarter were current smokers (*n* = 93, 25.8%). Physical activity levels indicated a substantial proportion of individuals with sedentary (*n* = 114, 31.7%) or light activity (*n* = 134, 37.2%) lifestyles. Moderate activity was reported by 105 (29.2%), while vigorous activity was minimal, reported by 7 (1.9%).

E-health literacy assessment

**Figure 1:**
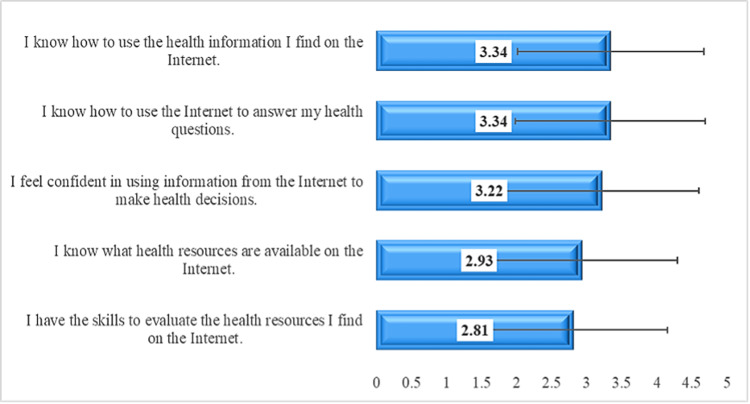
E-health literacy scores (eHEALS) among participants. E-health literacy items were rated on a 5-point Likert scale (1 = Strongly Disagree, 5 = Strongly Agree).

As shown in Figure 1, the overall mean e-health literacy score was 3.13 (standard deviation (SD) = 1.24) on a 5-point scale, indicating a moderate level of perceived e-health literacy among the participants. Participants reported higher perceived ability in "knowing how to use the Internet to answer my health questions" (*M* = 3.34, SD = 1.35) and "knowing how to use the health information I find on the Internet" (*M* = 3.34, SD = 1.33). However, lower mean scores were observed for "I have the skills to evaluate the health resources I find on the Internet" (*M* = 2.81, SD = 1.34) and "I know what health resources are available on the Internet" (*M* = 2.93, SD = 1.37). The perceived confidence in using internet information for health decisions was moderate (*M* = 3.22, SD = 1.38).

SDSCA

**Figure 2:**
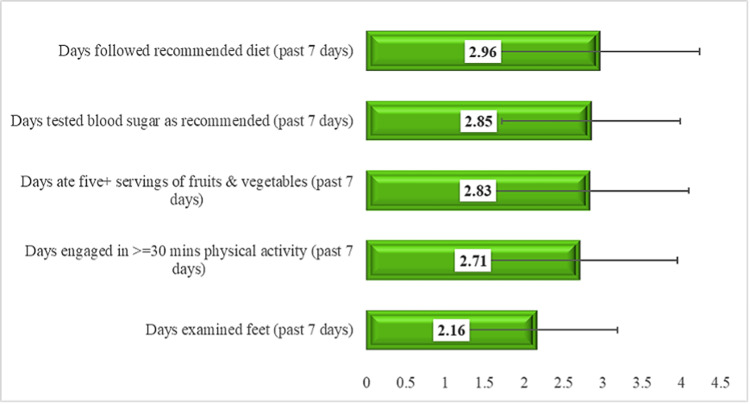
Summary of Diabetes Self-Care Activities (SDSCA) measure. Diabetes self-care activities items were rated on a scale representing days per week (1 = 0 days; 2 = 1-2 days; 3 = 3-4 days; 4 = 5-6 days or 7 days; 5 = 7 days where applicable).

As illustrated in Figure 2, the total mean score for diabetes self-care activities was 2.70 (SD = 0.937). This suggests a moderate level of adherence to self-care practices, generally falling around the three to four days per week range. Specifically, participants reported relatively better adherence to following their recommended diet (*M* = 2.96, SD = 1.26) and testing their blood sugar as recommended (*M* = 2.85, SD = 1.13, on a 1-4 scale). Eating five or more servings of fruits and vegetables (*M* = 2.83, SD = 1.26) and engaging in at least 30 minutes of physical activity (*M* = 2.71, SD = 1.24) showed slightly lower moderate adherence. Notably, foot care demonstrated the lowest mean score among all self-care activities (*M* = 2.16, SD = 1.02, on a 1-4 scale). 

Diabetes health outcomes

As presented in Table 2, the distribution of HbA1c levels reveals varied glycemic control among participants. Only a minority (*n* = 81, 22.5%) reported an HbA1c level below 7%, which is generally considered the target for good glycemic control in many individuals with diabetes. The largest proportion of participants had an HbA1c between 7% and 8% (*n* = 105, 29.2%). A significant portion also reported elevated HbA1c levels, with 73 (20.3%) between 8.1% and 9% and 74 (20.6%) above 9%. Furthermore, 26 (7.2%) participants reported not knowing their last HbA1c level. Regarding confidence in managing diabetes, the largest group reported feeling slightly confident (*n* = 104, 28.9%), followed closely by those feeling very confident (*n* = 102, 28.3%). Moderately confident accounted for 81 (22.5%) participants. However, a notable proportion (*n* = 73, 20.3%) indicated feeling not confident at all in managing their condition.

**Table 2:** Diabetes-related health outcomes among study participants.

Outcome measure	Category	n	%
Last recorded HbA1c level	<7%	81	22.5
	7%-8%	105	29.2
	8.1%-9%	73	20.3
	>9%	74	20.6
	I don’t know	26	7.2
Confidence in managing diabetes	Not confident	73	20.3
	Slightly confident	104	28.9
	Moderately confident	81	22.5
	Very confident	102	28.3

Challenges and barriers among study participants

As shown in Table 3, the most prevalent challenge reported by participants was difficulty understanding medical terms (*n* = 205, 56.9%). Closely following this, distrust in online information sources was identified by more than half of the participants (*n* = 196, 54.4%). Lack of internet access (*n* = 117, 32.5%) and difficulty navigating websites/apps (*n* = 114, 31.7%) were also significant barriers. A very small number of participants reported other specific challenges, such as conflicting opinions, busyness, or English language barriers, and only one participant indicated facing no difficulties at all (*n* = 1, 0.3%).

**Table 3:** Challenges in accessing online health information and barriers to adhering to a diabetes self-care routine among study participants. Participants could select multiple barriers. Percentages represent the proportion of the total sample (*N* = 360) endorsing each challenge or barrier. The total percentage may exceed 100%, as some participants reported more than one response.

Items		n	%
Challenge	Difficulty understanding medical terms	205	56.9
	Distrust of online information sources	196	54.4
	Lack of internet access	117	32.5
	Difficulty navigating websites/apps	114	31.7
	Conflicting opinions	1	0.3
	Busyness/Preoccupation	1	0.3
	English language	1	0.3
	No difficulty	1	0.3
Barrier	Lack of time	220	61.1
	Lack of knowledge	179	49.7
	Financial constraints	117	32.5
	Forgetfulness	99	27.5
	Physical limitations	26	7.2
	Habit of a certain lifestyle	1	0.3

Moreover, the most frequently cited barrier was lack of time (*n* = 220, 61.1%). Following closely, nearly half of the participants (*n* = 179, 49.7%) identified a lack of knowledge as a significant impediment to their self-care. Financial constraints were reported by 117 (32.5%) participants. Forgetfulness was also a common barrier, cited by 99 (27.5%) individuals. Physical limitations were a less frequent barrier, reported by 26 (7.2%) participants. Only one (0.3%) participant mentioned the habit of a certain lifestyle as another specified barrier.

The impact of e-health literacy on diabetes self-care activities

A hierarchical multiple linear regression analysis was conducted to investigate the impact of e-health literacy on diabetes self-care activities while controlling various sociodemographic and clinical factors, as shown in Table 4. The analysis was performed in two models. In the first model, only the Total E-health Literacy score was entered as a predictor. This model was statistically significant, *F*(1, 357) = 198.93, *P* < 0.001, and accounted for 35.8% of the variance in diabetes self-care activities (*R*² = 0.358, adjusted *R*² = 0.356). The Total E-health Literacy score was a significant positive predictor of SDSCA (*B* = 0.453, SE *B* = 0.032, *β* = 0.598, *P* < 0.001).

**Table 4:** Hierarchical multiple regression analysis predicting SDSCA from e-health literacy and sociodemographic/clinical factors. The dependent variable is SDSCA. Total E-health Literacy is the summed score of eHEALS items. *Significant at *P* < 0.05. **Significant at *P* < 0.01. *B*, unstandardized regression coefficient; SE *B*, standard error of the coefficient; β, standardized regression coefficient; eHEALS, E-health Literacy Scale; SDSCA, Summary of Diabetes Self-Care Activities

Predictor	B	SE *B*	β	t	P
Model 1					
Total E-health Literacy	0.453	0.032	0.598	14.104	<0.001
R²			0.358		
Adjusted *R*²			0.356		
*F*(1, 357)			198.93		<0.001
Model 2					
Total E-health Literacy	0.377	0.037	0.497	10.293	<0.001
Age	0.132	0.039	0.178	3.354	0.001**
Marital status	0.025	0.058	0.018	0.425	0.671
Gender	0.041	0.099	0.018	0.417	0.677
Educational level	0.093	0.048	0.101	1.918	0.056
Employment status	-0.034	0.044	-0.043	-0.789	0.431
Monthly household income	-0.044	0.048	-0.049	-0.918	0.359
Region of residence	0.142	0.086	0.063	1.648	0.100
Duration of diabetes	0.059	0.04	0.07	1.483	0.139
Type of diabetes treatment	-0.108	0.055	-0.083	-1.972	0.049*
Smoking status	-0.226	0.052	-0.178	-4.342	<0.001
Physical activity level	0.305	0.046	0.27	6.615	<0.001
*R*²			0.517		
Adjusted *R*²			0.501		
Δ*R*²			0.159		
*F*(12, 346)			30.91		<0.001

In the second model, sociodemographic and clinical variables (Age, Marital status, Gender, Educational level, Employment status, Monthly household income, Region of residence, Duration of diabetes, Type of diabetes treatment, Smoking status, and Physical activity level) were added. This model remained highly significant, *F*(12, 346) = 30.91, *P* < 0.001. The inclusion of these control variables significantly improved the model's explanatory power, with the full model accounting for 51.7% of the variance in SDSCA (*R*² = 0.517, adjusted *R*² = 0.501). The change in *R*² (Δ*R*²) from Model 1 to Model 2 was 0.159, indicating that the sociodemographic and clinical factors collectively explained an additional 15.9% of the variance in diabetes self-care activities beyond that explained by e-health literacy alone.

In Model 2, after controlling for the added variables, Total E-health Literacy remained a highly significant positive predictor of diabetes self-care activities (*B* = 0.377, SE *B* = 0.037, *β* = 0.497, *P* < 0.001). Among the control variables, several emerged as significant independent predictors of diabetes self-care activities. Age (*B* = 0.132, *β* = 0.178, *P* = 0.001): Older age was associated with higher self-care activities. This suggests that more experienced patients might be more diligent in their self-management. Type of diabetes treatment (*B* = -0.108, *β* = -0.083, *P* = 0.049): This negative coefficient implies that participants on insulin or combination therapy might engage in slightly fewer self-care activities compared to those on oral medications only. Smoking status (*B* = -0.226, *β* = -0.178, *P* < 0.001): Being a current or former smoker was significantly associated with lower self-care activities compared to never smoking. Physical activity level (*B* = 0.305, *β* = 0.270, *P* < 0.001): Higher levels of physical activity were strongly associated with increased self-care activities.

Key determinants of e-health literacy among patients with T2DM

As presented in Table 5, a significant difference in e-health literacy was observed based on gender (*t* = -3.04, *P* = 0.003). Male participants reported significantly higher mean e-health literacy scores (*M* = 3.23, SD = 1.221) compared to female participants (*M* = 2.756, SD = 1.228). Regarding age, there was a highly significant main effect of age on e-health literacy (*F* = 25.56, *P* < 0.001). Post hoc Bonferroni comparisons revealed a clear inverse relationship between age and e-health literacy. The youngest age group (30-39 years; *M* = 3.91, SD = 1.021) exhibited the highest e-health literacy, while the oldest group (60 years and above; *M* = 2.209, SD = 1.160) had the lowest. Specifically, participants aged 60 and above had significantly lower e-health literacy than all other age groups. 

**Table 5:** Differences in mean e-health literacy scores by sociodemographic and clinical characteristics. Values are presented as mean ± standard deviation. For gender, an independent samples t-test was used. For all other categorical variables with more than two groups, a one-way analysis of variance (ANOVA) was used, followed by Bonferroni post hoc tests. *Significant at *P* < 0.01. SD, standard deviation

Category	Sub-category	Mean ± SD	Test statistics	*P*-value	Post hoc significance (Bonferroni)
Gender	Male	3.23 ± 1.221	*t* = -3.04	0.003*	
	Female	2.75 ± 1.228			
Age (years)	<30	3.31 ± 0.920	*F* = 25.56	<0.001*	60+ < (Less than 30, 30-39, 40-49, 50-59); 50-59 < 30-39; 40-49 < 60+
	30-39	3.91 ± 1.021			
	40-49	3.49 ± 1.045			
	50-59	3.06 ± 1.173			
	60 and above	2.20 ± 1.160			
Marital status	Widowed	2.00 ± 1.117	*F* = 9.34	<0.001*	Widowed < (Single, Married)
	Single	3.39 ± 0.894			
	Married	3.21 ± 1.258			
	Divorced	2.75 ± 1.186			
Educational level	No formal education	2.860 ± 1.113	*F* = 26.23	<0.001*	Postgraduate > (No formal, Primary, Secondary, College/University); College/University > (Primary, Secondary); Secondary > (Primary)
	Primary education	2.157 ± 1.064			
	Secondary education	2.494 ± 1.134			
	College/University degree	3.199 ± 1.126			
	Postgraduate education	4.228 ± 0.917			
Employment status	Employed (full-time)	3.536 ± 1.159	*F* = 18.20	<0.001*	Employed (full-time) > (Employed part-time, Retired, Unemployed)
	Employed (part-time)	2.835 ± 1.068			
	Retired	2.580 ± 1.163			
	Unemployed	2.623 ± 1.176			
Monthly household income	<3,000 SAR	2.443 ± 1.209	*F* = 27.02	<0.001*	Above 10,000 > (all other income groups); 5,001-10,000 > (<3,000, 3,000-5,000)
	3,000-5,000 SAR	2.438 ± 1.119			
	5,001-10,000 SAR	2.793 ± 1.094			
	Above 10,000 SAR	3.642 ± 1.111			
Region of residence	Suburban area	2.367 ± 1.202	*F* = 16.37	<0.001*	Makkah City Center > (Suburban, Rural)
	Makkah City Center	3.294 ± 1.191			
	Rural area	2.320 ± 1.077			
Duration of diabetes	<1 year	3.582 ± 0.985	*F* = 15.54	<0.001*	>10 years < (<1 year, 1-5 years, 6-10 years)
	1-5 years	3.532 ± 1.190			
	6-10 years	3.236 ± 1.048			
	>10 years	2.617 ± 1.272			
Type of diabetes treatment	Oral medications only	3.437 ± 1.190	*F* = 26.44	<0.001*	Oral medications only > (Insulin only); Combination > (Insulin only)
	Insulin only	2.389 ± 1.190			
	Combination	3.117 ± 0.911			
Smoking status	Never smoked	3.392 ± 1.203	*F* = 14.83	<0.001*	Never smoked > (Current smoker)
	Current smoker	2.596 ± 1.304			
	Former smoker	3.004 ± 0.897			
Physical activity level	Sedentary	2.655 ± 1.357	*F* = 18.31	<0.001*	Vigorous > (Sedentary, Light); Moderate > (Sedentary, Light)
	Light activity	3.004 ± 1.087			
	Moderate activity	3.722 ± 1.014			
	Vigorous activity	4.257 ± 0.763			

Marital status significantly influenced e-health literacy (*F* = 9.34, *P* < 0.001). Post hoc tests indicated that widowed participants (*M* = 2.00, SD = 1.117) had significantly lower e-health literacy scores compared to both single (*M* = 3.39, SD = 0.894) and married (*M* = 3.21, SD = 1.258) individuals. There were no significant differences between single, married, and divorced groups. Educational attainment was a strong determinant of e-health literacy (*F* = 26.23, *P* < 0.001). A positive correlation was observed, where higher educational levels were associated with greater e-health literacy. Participants with postgraduate education (*M* = 4.22, SD = 0.917) reported the highest scores, significantly higher than all other education groups. Similarly, those with a college/university degree (*M* = 3.19, SD = 1.126) had significantly higher e-health literacy than those with primary or secondary education. 

Employment status also significantly impacted e-health literacy (*F* = 18.20, *P* < 0.001). Full-time employed individuals (*M* = 3.53, SD = 1.159) demonstrated significantly higher e-health literacy compared to part-time employed (*M* = 2.83, SD = 1.068), retired (*M* = 2.58, SD = 1.163), and unemployed (*M* = 2.62, SD = 1.176) participants. Monthly household income showed a highly significant association with e-health literacy (*F* = 27.02, *P* < 0.001). Participants in the highest income bracket (above 10,000 SAR; *M* = 3.64, SD = 1.111) had significantly greater e-health literacy than all other income groups. Moreover, those earning 5,001-10,000 SAR (*M* = 2.79, SD = 1.094) had higher e-health literacy than those earning less than 3,000 SAR or 3,000-5,000 SAR. 

On the other hand, e-health literacy varied significantly by region of residence (*F* = 16.37, *P* < 0.001). Participants residing in City Center (*M* = 3.29, SD = 1.191) reported significantly higher e-health literacy compared to those in suburban areas (*M* = 2.36, SD = 1.202) and rural areas (*M* = 2.32, SD = 1.077). Duration of diabetes had a significant effect on e-health literacy (*F* = 15.54, *P* < 0.001). Participants with a shorter duration of diabetes (<1 year: *M* = 3.58, SD = 0.985; 1-5 years: *M* = 3.53, SD = 1.190) generally exhibited higher e-health literacy compared to those with a longer duration (>10 years: *M* = 2.61, SD = 1.272). Significant differences in e-health literacy were found across different types of diabetes treatment (*F* = 26.44, *P* < 0.001). Individuals on oral medications only (*M* = 3.43, SD = 1.190) demonstrated significantly higher e-health literacy than those on insulin only (*M* = 2.38, SD = 1.190). Participants on a combination of oral medications and insulin (*M* = 3.11, SD = 0.911) also had significantly higher e-health literacy than those on insulin only.

In addition, smoking status significantly influenced e-health literacy (*F* = 14.83, *P* < 0.001). Non-smokers (*M* = 3.39, SD = 1.203) reported significantly higher e-health literacy than current smokers (*M* = 2.59, SD = 1.304). Finally, physical activity level was significantly associated with e-health literacy (*F* = 18.31, *P* < 0.001). A clear gradient was observed, with higher physical activity correlating with greater e-health literacy. Participants engaging in vigorous activity (*M* = 4.25, SD = 0.763) and moderate activity (*M* = 3.72, SD = 1.014) had significantly higher e-health literacy than those who were sedentary (*M* = 2.65, SD = 1.357) or engaged in light activity (*M* = 3.00, SD = 1.087). 

The influence of e-health literacy on diabetes-related health outcomes

A univariate binary logistic regression analysis was conducted to examine the association between total e-health literacy scores and the likelihood of achieving controlled HbA1c levels among patients with T2DM (Table 6). The overall model was statistically significant (*χ*²(1) = 69.08, *P* < 0.001), indicating that total e-health literacy significantly predicts the likelihood of having controlled HbA1c. The model explained a moderate amount of variance in HbA1c control, with a Nagelkerke *R*² of 0.279. This suggests that approximately 27.9% of the variability in HbA1c control can be accounted for by e-health literacy in this univariate model. The model correctly classified 81.7% of cases overall. More specifically, it correctly classified 92.9% of the uncontrolled HbA1c cases and 46.9% of the controlled HbA1c cases. The results table shows that total e-health literacy was a significant positive predictor of controlled HbA1c, with an OR of 2.985 (95% confidence interval (CI) 2.178-4.092). This indicates that for every one-unit increase in the total e-health literacy score, the odds of having controlled HbA1c (HbA1c < 7%) are approximately 2.985 times higher, or nearly three times greater, when compared to the odds of having uncontrolled HbA1c.

**Table 6:** Univariate logistic regression analysis predicting controlled HbA1c. The dependent variable is dichotomized HbA1c (1 = Controlled HbA1c, 0 = Uncontrolled HbA1c). Model statistics: *χ*²(1) = 69.08, *P* < 0.001; Nagelkerke *R*² = 0.279. Overall percentage correctly classified = 81.7%. *Significant at *P* < 0.01. *B*, unstandardized logistic regression coefficient; SE, standard error; OR, odds ratio; CI, confidence interval.

Predictor	OR	95% CI for OR	Wald	df	P
Total E-health Literacy	2.985	[2.178, 4.092]	46.196	1	<0.001*
Constant	0.007		62.454	1	<0.001

Finally, an ordinal logistic regression was performed to examine the association between total e-health literacy score and participants' confidence in managing their diabetes (Table 7). The final model was significant (*χ*²(1) = 190.68, *P* < 0.001). This indicates that e-health literacy is a significant predictor of confidence in managing diabetes. The model explained a substantial proportion of the variance in confidence, with a Nagelkerke pseudo *R*² value of 0.440, suggesting that approximately 44.0% of the variability in diabetes management confidence can be accounted for by e-health literacy. The Total E-health Literacy was statistically significant (*B* = 1.297, SE = 0.106, Wald *χ*²(1) = 151.06, *P* < 0.001). The OR was 3.658, with a 95% CI (2.974-4.500). This indicates that for every one-unit increase in a participant's total e-health literacy score, the odds of moving to a higher category of confidence in managing diabetes (e.g., from *Not confident* to *Slightly confident*, or from *Slightly confident* to *Moderately confident*, etc.) are approximately 3.66 times greater.

**Table 7:** Ordinal regression analysis predicting confidence in managing diabetes from total e-health literacy. Dependent variables: Confidence in managing diabetes (1 = Not confident, 2 = Slightly confident, 3 = Moderately confident, 4 = Very confident). The threshold parameters represent the cut points on the latent logit scale for cumulative probabilities. Link function: Logit. Model Fit: Initial -2 Log Likelihood = 429.79, Final -2 Log Likelihood = 239.11; *χ*²(1) = 190.68, *P* < 0.001. Pseudo *R*²: Cox and Snell = 0.412, Nagelkerke = 0.440, McFadden = 0.193. Goodness-of-fit (Pearson): *χ*²(59) = 135.62, *P* < 0.001; goodness-of-fit (deviance): *χ*²(59) = 122.17, *P* < 0.001. *Results that are statistically significant at *P* < 0.01. *B*, unstandardized logistic regression coefficient; SE, standard error; OR, odds ratio; CI, confidence interval

Predictor	B	SE	Wald	P	OR	95% CI for OR
Location parameter						
Total E-health Literacy	1.297	0.106	151.061	<0.001*	3.658	2.974-4.500
Threshold parameters						
Not confident	2.077	0.3	48.054	<0.001*		
Slightly confident	4.064	0.36	127.096	<0.001*		
Moderately confident	5.435	0.403	181.596	<0.001*		

## Discussion

This study explored the significant impact of e-health literacy on diabetes self-care activities among individuals with T2DM in Saudi Arabia. The findings align with previous research while revealing unique sociodemographic disparities and barriers to digital health adoption. 

The overall mean SDSCA score of 2.70, which reflects approximately three to four days per week of adherence to recommended self-care activities [4,12,18], indicates suboptimal diabetes self-management when compared to clinical guidelines. For instance, best practices recommend daily self-monitoring of blood glucose, consistent dietary adherence, regular physical activity (ideally most days of the week), and daily foot care [12,18]. However, participants reported lower adherence, especially in foot care (*M* = 2.16), suggesting important gaps in routine practices. These findings are clinically significant, as inadequate self-care is associated with poorer glycemic control and increased risk of complications [12]. This underscores the need for targeted interventions to improve specific domains of diabetes self-care.

The findings of this study highlight a significant positive relationship between e-health literacy and diabetes self-care activities among individuals with T2DM in Makkah City, Saudi Arabia. The results align with previous research, which has consistently demonstrated that higher e-health literacy is associated with better self-care practices, including medication adherence, dietary management, and physical activity [4,10]. In this study, participants with higher e-health literacy scores reported greater adherence to self-care activities, such as monitoring blood glucose levels and following dietary recommendations. This corroborates the findings of Guo et al. [4], who found that mobile e-health literacy significantly improved self-care behaviors and glycemic control among Taiwanese patients with T2DM. Similarly, Lee et al. [10] emphasized the role of e-health literacy in enhancing self-management through improved access to and utilization of digital health resources. However, the moderate mean e-health literacy score (3.13 out of 5) observed in this study suggests room for improvement, particularly in critical evaluation skills and awareness of available health resources. This finding contrasts with studies conducted in high-income countries, where e-health literacy levels tend to be higher due to greater access to digital infrastructure and health education programs [19]. The lower scores in evaluating online health resources and identifying reliable information sources may reflect broader challenges in health literacy, as noted by Berkman et al. [14], who highlighted the adverse effects of limited health literacy on health outcomes. This underscores the need for targeted interventions to enhance both digital and health literacy skills among diabetic patients in Saudi Arabia.

The study also revealed disparities in e-health literacy based on socio-demographic factors. For instance, male participants, younger individuals, and those with higher education and income levels reported significantly higher e-health literacy scores. These findings are consistent with previous research indicating that age, education, and socioeconomic status are key determinants of e-health literacy [17,19]. The inverse relationship between age and e-health literacy is particularly noteworthy, as older adults often face greater challenges in adopting digital technologies [20]. This demographic gap highlights the importance of designing age-appropriate e-health interventions to ensure inclusivity and accessibility for all patient groups.

The positive association between e-health literacy and glycemic control (HbA1c levels) further reinforces the value of digital health resources in diabetes management. Participants with higher e-health literacy were nearly three times more likely to achieve controlled HbA1c levels, a finding that aligns with studies by Guo et al. [4] and Mashi et al. [21]. The latter study also identified health literacy as a critical factor in glycemic control among Saudi patients. However, the relatively low proportion of participants (22.5%) with controlled HbA1c levels suggests that e-health literacy alone may not suffice to address all barriers to effective diabetes management. Other factors, such as access to healthcare, social support, and financial constraints, must also be considered [22,23].

The study identified several barriers to e-health literacy and self-care, including difficulty understanding medical terms, distrust in online information, and lack of time. These challenges mirror those reported in other studies, where patients often struggle with the complexity of health information and the reliability of online sources [24,25]. The prevalence of distrust in online health information is particularly concerning, as it may deter patients from utilizing valuable digital resources. This issue has been addressed in interventions that combine e-health literacy training with critical appraisal skills, such as the program developed by Guo et al. [4], which significantly improved participants' ability to evaluate and use online health information effectively. Another critical finding is the role of social support in mediating the relationship between e-health literacy and self-care activities. While this study did not explicitly measure social support, previous research has shown that emotional and informational support from family and healthcare providers can enhance patients' confidence and ability to manage their condition [10,22]. Future interventions have to incorporate social support mechanisms, such as peer-led education or family-involved training, to amplify the benefits of e-health literacy programs.

The study's focus on primary healthcare centers in Makkah provides valuable insights into the urban context of diabetes management in Saudi Arabia. However, the underrepresentation of rural populations and the predominance of male participants (78.3%) may limit the generalizability of the findings as well as the dominance of female, lower socioeconomic populations, and reliance on self-reported measures, which may introduce recall and social desirability biases. The study included only participants who had accessed online health information, which may have introduced selection bias and inflated overall e-health literacy. These limitations are consistent with challenges noted in other studies conducted in similar settings [2,26]. Future research should aim to include more diverse populations to ensure broader applicability of the results. While the questionnaire underwent translation and back-translation by bilingual experts and was reviewed by a panel of professionals in diabetes care, e-health, and public health, psychometric re-validation of the translated scales was not conducted formally. Thus, future studies should incorporate full psychometric testing to ensure reliability and validity in the target population. Finally, unmeasured confounders (e.g., healthcare access, cultural factors) may influence outcomes. Future research should address these gaps with longitudinal, more diverse samples and objective measures.

## Conclusions

In conclusion, this study contributes to the growing body of evidence supporting the role of e-health literacy in improving diabetes self-care and glycemic control. The findings highlight the need for multifaceted interventions that address not only digital skills but also health literacy, social support, and systemic barriers. By integrating e-health literacy training into diabetes education programs and tailoring interventions to meet the needs of diverse patient groups, healthcare providers can empower individuals with T2DM to take an active role in managing their condition. As digital health technologies continue to evolve, ongoing research and innovation will be essential to maximize their potential in improving health outcomes for diabetic patients worldwide.

To enhance e-health literacy among vulnerable groups, implement mobile apps with visual/audio aids, WhatsApp-based education, and interactive kiosks in clinics. Offer community-based digital literacy workshops and peer support programs, and integrate e-health training into routine care. Encourage providers to recommend trusted digital tools. To prevent digital divide-related disparities, use hybrid approaches, provide non-digital alternatives, and consider subsidies for devices or internet access to ensure inclusive and equitable healthcare access.
